# On the Investigation of Some of the Nervous Disorders of the Heart

**Published:** 1897-10-16

**Authors:** Arthur Ernest Sansom

**Affiliations:** President of the Society


### Medical Progress and Hospital Clinics.

[*The Editor will be glad to receive offers of co-operation and contributions from members of the profession. All letters should be addressed to* The Editor, at the Office, 28 & 29, Southampton Street, Strand, London, W.C.]

### ON THE INVESTIGATION OF SOME OF THE NERVOUS DISORDERS OF THE HEART.

Being the Opening Address of the 125th Session of the Medical Society of London. By Arthur Ernest Sansom, M.D., F.R.C.P., President of the Society.

After some appropriate introductory remarks, Dr. Sansom said: I have come to the conclusion that perturbations of the heart's rhythm, whether in the sense of tachycardia, or of arhythmia, or of brachycardia, are essentially independent of any structural disease of the heart and of its valves, and are entirely due to causes involving the nervous mechanism of the cardiac reflex. In a great majority of such cases careful physical examination during life reveals no trustworthy evidence of structural disease, and in some of the few in which death has occurred, and has been followed by autopsy, no organic changes in the heart have been noted. I conclude that when structural disease of the heart has been observed in the subjects of perturbed rhythm, either the latter has been a coincidence, a superadded phenomenon, or the organic cardiac changes have been secondary to the disorder of the nerve mechanism. For instance, I have seen well-marked Graves's disease supervene upon chronic valvular disease—the result of antecedent rheumatic endocarditis; and I have observed dilatation of the heart with mitral incompetence to occur in the course of Graves's disease when there has been no reason to suspect any intercurrent malady. In the latter case I have found that the signs of structural disease of the heart have borne no proportion to the degree of cardiac tumult.

The subject of a very rapid heart (120 to 160, such rate being manifested for many months) may never show signs of cardiac dilatation, nor of structural change, whilst one in whom there is only a moderate tachycardia (100 and under) may manifest the advent of undoubted dilatation of the left ventricle with valvular incompetence.

If we accept it as a working hypothesis that serious disturbances of the action of the heart and serious symptoms interfering with the sense of its well-being may occur without morbid changes, detectable by physical examination, in the heart itself, there is a very important corollary, viz., that it is necessary in every case that, in addition to the employment of the ordinary methods of physical diagnosis for the discovery of organic changes within the heart, there should be a careful investigation of the nervous system, more especially in its relation with the cardiac reflex. On this point I propose to suggest a scheme of examination, and to offer a few hints. In the first place I would urge the importance of keeping the patient who is to be examined as tranquil as possible. In the case of a patient in bed I have nothing to say; the examination will, of course, be conducted in such way as to cause as little disturbance as possible. In the case of one who comes for consultation in ordinary out-of-door apparel, I suggest that he should sit down and not attempt to disrobe. It is easy enough to excite a patient, but it is not so easy to keep him or her quiet. I have known the rate of the heart-beats to be increased in an extraordinary degree by a mere change from the sitting or recumbent to the standing position. One who peels off his clothes in a state of excitement may not only cause for himself an abnormally rapid pulse, but may, I think, manufacture a murmur near the heart's apex which may lead to erroneous inferences. I suggest, therefore, that the examination should be commenced when the patient is in the sitting position, the ordinary clothing not being removed.

I. Amongst the most important are the signs observed by inspection of the eyes and eyelids. Any protuberance of the eyeball will, of course, be observed, and its relation with the condition of the thyroid and the rate of the heart's pulsation recorded. Any retraction of the upper eyelids towards the margin of the orbit must also be noted, for this is a sign of considerable importance. It often exaggerates the prominence of a bulging globe, and sometimes I have seen that it produces the semblances of an exophthalmos when there is really no prominence of the globe.

I have observed another sign in regard to the upper eyelids in many cases of tachycardia. When the patient has been told to close the eyelids gently the upper lid has manifested a very marked and unusual tremor. I have observed this sign in the early stages and also in the recovering stages of Graves's disease. Von Graefe's sign and Dalrymple's (eyelid retraction) often co-exist, but in some cases the one is observed and not the other.

I have noticed (as recorded by Dr. Dixon Mann) that in some cases the normal balance of the muscles of the globe is somewhat perverted, so that one eye appears to be on a lower level than the other (Trans. Ophthal. Soc., 1886).

I have also noted in some instances a decided inequality of the pupils—of course unexplained by organic causes—which became restored to the normal when the other disorders of function ceased. When these signs exist, especially in combination, I think they have an important significance. In the great majority of cases they are associated with heart disturbance or heart distress. The most frequent form of disorder is tachycardia—the rapid heart—persistent or paroxysmal. This has long been recognised; the association of the eye and eyelid phenomena with Graves's disease, of which tachycardia is so marked a feature, has been fully acknowledged.

Less frequently the association is with extreme irregularity of the heart's action; and such irregularity, rather than rapidity, I have found in some cases of typical Graves's disease. I have observed well marked Von Graefe's sign in a man aged 49, whose pulse was only 68, but showed very marked irregularity, and in whom there was much subjective heart distress. In some of the cases in which I have observed the signs noted, there were great variations of rate in the heart-beat—a state which may perhaps be termed irritable heart. For instance, in a man of 48, manifesting slight exophthalmos and inequality of pupils, the heart-rate was 76 in the sitting position, 84 in the standing, and then a sudden rise (without any evidence of discomfort) to 114. In other cases, though there have been no perturbations of rate and rhythm, the patients who have shown well-marked eye and eye-lid signs have manifested symptoms of distress referred to the heart. It is no doubt correct that well-marked eyelid phenomena may occur (though rarely) in those who show no cardiac sign nor symptoms. Nevertheless, I cannot doubt that the signs I have mentioned are of considerable practical significance, for they indicate very strongly the probability that the patient who manifests them will present signs of disorder or distress of the heart.

II. It is important that the auditory mechanism and the pharyngeal tract be explored, for from data which I have already published (Med. Soc. Trans., Vol. XVI. p. 107) I have convinced myself that irregularity of the heart is sometimes the result of a reflex occasioned by a morbid irritation of some part of the naso-pharyngeal tract or of the auditory mechanism.

III. The pulse at the wrist should be carefully explored by palpation, and its lessons supplemented and controlled by a determination of the rate and character of the heart's pulsations as determined by auscultation. Such auscultations should at first be practised when the patient is sitting quietly—the clothing as yet unremoved. Much valuable evidence, notably the accentuation of the second sound and its predominance in the aortic or pulmonic area, is obtained by this preliminary method of auscultation.

IV. The existence and site of muscular tremors should be observed and noted. If fine tremors of the muscles of the trunk, the limbs, or the face are observed, there is a probability of a disorder of the nervous mechanism of the heart of the nature of Graves's disease.

V. The clothing being removed, the chest is of course to be examined by the ordinary means of physical diagnosis.

In regard to the nervous disorders of the heart, the most important points are the determination and the significance of dilatation of the heart, and the characters and diagnostic indications of a systolic murmur heard at or near the apex. Extreme dilatation of the heart may occur from causes affecting the nervous elements, such as neuritis of the vagi, and yet the normal conditions may return in the course of a fortnight. The bulk of the heart in some cases varies considerably in the course of twenty-four hours.

A systolic murmur heard at or near the apex of the heart is sometimes erroneously interpreted as the index of structural disease. It may certainly be entirely independent thereof, and only an indication of nervous disorder. In such cases it is of the first importance to determine whether or no there are persistent changes in the bulk of the various chambers, or in the sounds which indicate overstrain in the circuits. A loud second sound over the pulmonary artery means structural disease. On the other hand, in the case of a systolic apical murmur due to neurosis, the heart is not dilated, and in the great majority of cases is smaller than the normal.

A systolic murmur which is independent of structural disease seldom attains its maximum audibility at the exact apex, but slightly to the right, to the left, or above this point. It is usually soft, and does not replace the first sound.

It does not occupy the whole, but a middle period of the systole (it is meso-systolic). It is much influenced by the movements of respiration. As a rule, it is intensified both during expiration and inspiration (especially the latter), but it often becomes inaudible at the end of an expiration. If, therefore, rhythmical crescendo and diminuendo in the sound of the murmur are heard during the respiratory acts, the murmur is probably not due to organic change.

It is important that auscultation should be practised, not only in the erect or sitting, but also in the recumbent position of the patient. Very frequently a murmur is very audible in the recumbent position, but disappears when the sitting or erect position is assumed. In such case a confident opinion may be given that there is no structural disease. In a minority of cases the rule is reversed, and a murmur which is not detected in a patient in the recumbent position becomes audible when the erect or sitting posture is assumed.

Potain has made a most valuable and elaborate study of these, which he calls cardio-pulmonary murmurs; but, with the highest appreciation of his researches, I think that a more probable explanation of the murmur is to be found in a derangement of the nervous mechanism of the cardiac systole. The ventricular systole is a complex act—a correlated sequence of muscular contractions—as shown by the remarks of Roy and Adami, of Ludwig and Hesse. It is not difficult to realise that these may be disturbed, and may continue so disarranged for protracted periods, the effect of such disarrangement being a slight mitral reflex, harmless and transitory, or abnormal vibration communicated to certain portions of the structure. Whatever the proximate cause, the ultimate is a disturbance of the nervous mechanism of the heart. My object has been to urge the importance of an investigation into the nervous condition of the heart which should go *pari passu* with the use of the ordinary means of physical diagnosis.

